# Rationale and Design of a Pharmacist-led Intervention for the Risk-Based Prevention of Heart Failure: The FIT-HF Pilot Study

**DOI:** 10.3389/fcvm.2021.785109

**Published:** 2021-11-29

**Authors:** Michael C. Wang, Bridget Dolan, Benjamin H. Freed, Lourdes Vega, Nikola Markoski, Amy E. Wainright, Bonnie Kane, Laura E. Seegmiller, Katharine Harrington, Alana A. Lewis, Sanjiv J. Shah, Clyde W. Yancy, Ian J. Neeland, Hongyan Ning, Donald M. Lloyd-Jones, Sadiya S. Khan

**Affiliations:** ^1^Department of Preventive Medicine, Northwestern University Feinberg School of Medicine, Chicago, IL, United States; ^2^Department of Pharmacy, Northwestern Memorial Hospital, Chicago, IL, United States; ^3^Division of Cardiology, Northwestern University Feinberg School of Medicine, Chicago, IL, United States; ^4^Department of Medicine, University Hospitals Cleveland Medical Center, Cleveland, OH, United States; ^5^Case Western Reserve University School of Medicine, Cleveland, OH, United States

**Keywords:** heart failure, primary prevention, pharmacist, risk prediction, natriuretic peptides

## Abstract

**Background:** Given rising morbidity, mortality, and costs due to heart failure (HF), new approaches for prevention are needed. A quantitative risk-based strategy, in line with established guidelines for atherosclerotic cardiovascular disease prevention, may efficiently select patients most likely to benefit from intensification of preventive care, but a risk-based strategy has not yet been applied to HF prevention.

**Methods and Results:** The Feasibility of the Implementation of Tools for Heart Failure Risk Prediction (FIT-HF) pilot study will enroll 100 participants free of cardiovascular disease who receive primary care at a single integrated health system and have a 10-year predicted risk of HF of ≥5% based on the previously validated Pooled Cohort equations to Prevent Heart Failure. All participants will complete a health and lifestyle questionnaire and undergo cardiac biomarker (B-type natriuretic peptide [BNP] and high-sensitivity cardiac troponin I [hs-cTn]) and echocardiography screening at baseline and 1-year follow-up. Participants will be randomized 1:1 to either a pharmacist-led intervention or usual care for 1 year. Participants in the intervention arm will undergo consultation with a pharmacist operating under a collaborative practice agreement with a supervising cardiologist. The pharmacist will perform lifestyle counseling and recommend initiation or intensification of therapies to optimize risk factor (hypertension, diabetes, and cholesterol) management according to the most recent clinical practice guidelines. The primary outcome is change in BNP at 1-year, and secondary and exploratory outcomes include changes in hs-cTn, risk factor levels, and cardiac mechanics at follow-up. Feasibility will be examined by monitoring retention rates.

**Conclusions:** The FIT-HF pilot study will offer insight into the feasibility of a strategy of quantitative risk-based enrollment into a pharmacist-led prevention program to reduce heart failure risk.

**Clinical Trial Registration:** https://clinicaltrials.gov/ct2/show/NCT04684264

## Background

Although overall cardiovascular disease mortality has declined 3-fold over the past five decades (1), this has been largely driven by improvement in ischemic heart disease mortality rates (2). Advances in the prevention of ischemic heart disease have now been offset by increasing mortality related to heart failure (HF), resulting in stagnation in overall cardiovascular disease mortality rates since 2011 (3). HF affects an estimated six million American adults, a prevalence which is expected to increase nearly 50% by 2030, and is responsible for ~1 million hospitalizations and two million physician office visits annually (4).

Given the large and rising morbidity, mortality, and costs of HF, evidence-based and generalizable prevention strategies, which currently exist for atherosclerotic cardiovascular disease (ASCVD) (5) but not HF, are needed. Two important questions in the design of a potential HF prevention strategy include (1) how to assess patients' individual risk for HF and (2) how to intensify prevention efforts in patients identified as high risk.

### Heart Failure Risk Assessment

The 2017 focused update of the American College of Cardiology/American Heart Association (ACC/AHA) Guideline for the Management of HF highlights the potential role of natriuretic peptide biomarker screening, followed by team-based care, in those at risk for HF (6). This guideline recommendation was informed by two randomized controlled trials, the STOP-HF trial and the PONTIAC trial, which demonstrated that referral to collaborative cardiology care based on natriuretic peptide value cutoffs in patients at high cardiovascular risk may prevent HF (7, 8). However, in STOP-HF, only 27% of patients had a BNP > 50 pg/mL, the threshold for referral to cardiology. In addition, a risk factor-based approach that treats all risk factors equally (as in STOP-HF) or requires the presence of a particular risk factor such as diabetes (as in PONTIAC) may miss some patients at high risk due to elevations below treatment thresholds in multiple risk factors and may over-sample some patients at low risk, such as younger adults with a single risk factor. In contrast, multivariable risk prediction equations for HF, such as the Pooled Cohort equations to Prevent HF (PCP-HF) (9), that incorporate clinical measures readily available in primary care settings may improve identification of those at risk for HF who may merit more intensive prevention strategies.

### Pharmacist-led Intensification of Preventive Care

Efforts to intensify preventive care have been implemented with a variety of healthcare and non-healthcare team members such as pharmacists (10, 11), nurses (12, 13), and community healthcare workers (14), and through leveraging digital tools such as web-based portals (15) and remote monitoring (16). Several prior reports suggest a potential benefit of involvement of these team members in strategies for hypertension or diabetes control (11, 13–16); for example, a trial of a nurse-led clinic for patients with diabetes showed that, at 1 year, 37.2% of patients in the intervention met their cholesterol and/or blood pressure target compared with 30.7% of patients receiving usual care (OR 1.37 [95% CI: 1.11, 1.69]) (13). Pharmacist-led interventions have unique advantages, as pharmacists are experts in the medications that achieve core targets of primary prevention, including blood pressure, glucose, and lipid lowering; pharmacists are well-positioned to counsel patients about sodium-glucose cotransporter-2 (SGLT-2) inhibitors, one of the most promising recent medications for the prevention of heart failure (17); and pharmacists may, under collaborative practice agreements with supervising physicians, directly prescribe and titrate medications for patients. In addition, pharmacists may be deployed in communities and have a potent effect on preventive care for underserved populations, as was recently demonstrated in a trial of a pharmacist-led intervention for blood pressure reduction in barbershops in predominantly Black communities (18).

### Rationale of Risk-Based Heart Failure Prevention

It would be neither feasible nor cost-effective to perform biomarker screening or intensify preventive efforts in the entire population. Risk-based prevention strategies, in which the intensity of prevention efforts are tailored to the predicted risk of disease based on a comprehensive risk factor profile, maximize the potential benefits of preventive strategies and are well-established in atherosclerotic cardiovascular disease (ASCVD) (19–21). However, a quantitative risk-based approach has not yet been applied to the primary prevention of heart failure and requires further investigation with regard to the feasibility and efficacy of such an approach. Therefore, in this report we describe the design of a pilot study of a pharmacist-led intervention program for risk-based prevention of HF, the Feasibility of the Implementation of Tools for Heart Failure Risk Prediction (FIT-HF) Study. The FIT-HF Study will enroll 100 patients free of any type of cardiovascular disease with a predicted 10-year risk of developing heart failure, based upon the PCP-HF, (9) of 5% or higher. Patients will undergo BNP, hs-cTn, and echocardiogram testing at baseline and will be randomized to a pharmacist-led intervention or usual care. Successful completion of this pilot study will demonstrate feasibility of the proposed strategy and generate preliminary data to support the design of a subsequent, well-powered trial evaluating clinical event outcomes.

## Study Design

### Eligible Study Population

The FIT-HF study will enroll 100 participants who receive primary care at a single integrated academic healthcare center, Northwestern Medicine. Eligible participants will be aged 30–79 years and have at least one internal medicine visit in the past year, at least two such visits in the past 5 years, and cholesterol and glucose levels measured within the past 5 years. In addition, using the most recently available data in the electronic health record (EHR), participants will have a predicted 10-year risk of developing HF of at least 5% based on the PCP-HF model (9). The variables in the PCP-HF model are age, sex, race, smoking, body mass index, systolic blood pressure, hypertension treatment, fasting glucose, diabetes treatment, total cholesterol, high density lipoprotein cholesterol, and QRS duration. Because real-world glucose measurements are often non-fasting, and QRS duration is not routinely clinically available, a version of the PCP-HF equations calibrated to random glucose values and excluding QRS duration will be used (22). The 5% cutoff of 10-year predicted HF risk represents approximately the top quintile of risk among US adults (23). Participants are ineligible if they have a history of cardiovascular disease (by EHR diagnosis code or self-report), signs or symptoms of HF, a current pregnancy, an estimated glomerular filtration rate <45 mL/min/1.73 m^2^ using the CKD-EPI (2009) formula either prior to or during the baseline exam, or stage three or four cancer. Detailed inclusion and exclusion criteria are available in the Supplementary Methods.

### Study Recruitment

Recruitment will be accomplished leveraging the robust Northwestern Medicine Enterprise Data Warehouse (NMEDW), which is a comprehensive and integrated repository of all clinical, administrative, and research data sources on the campus. Briefly, the NMEDW currently stores observations on more than 7.1 million distinct patients and is updated in real time with data elements from 142 separate sources, including electronic health records, pathology data from the hospital and research laboratories, biomarker data from research databases and research transactional data from the Northwestern Institutional Review Board and other institutional systems. The NMEDW-based algorithm has been customized to identify all patients meeting study eligibility criteria based on existing data in their electronic health record and automatically excludes patients who opted out of being contacted for research studies, thus allowing for targeted and highly efficient recruitment of patients. The baseline characteristics of patients in the recruitment report are listed in Table 1, which identified 13,059 potentially eligible patients with a mean (standard deviation) age of 66.6 (8.5) years. The time frame for full enrollment is 1 year, with the final participant expected to complete the protocol 2 years after the beginning of the study.

**Table 1:** Baseline characteristics of patients potentially eligible for recruitment.

***N***	**13,059**
Age (years), mean (SD)	66.6 (8.5)
**Gender**, ***N*** **(%)**	
Female	6,040 (46.3%)
Male	7,019 (53.7%)
**Self-reported race**, ***N*** **(%)**	
White	8,832 (67.6%)
Black or African American	2,210 (16.9%)
Asian	450 (3.4%)
Other or Declined	1,567 (12.0%)
Body mass index (kg/m^2^), median (IQR)	29.7 (26.1, 34.6)
Systolic blood pressure (mmHg), mean (SD)	136.1 (17.2)
Diastolic blood pressure (mmHg), mean (SD)	78.3 (10.0)
Glucose (mg/dL), mean (SD)	116.3 (42.4)
Total cholesterol (mg/dL), mean (SD)	181.3 (40.5)
HDL cholesterol (mg/dL), mean (SD)	53.8 (15.7)
LDL cholesterol (mg/dL), mean (SD)	101.6 (36.3)
**Medication use**, ***N*** **(%)**	
Antihypertensive	10,594 (81.1%)
Antidiabetic	2,919 (22.4%)
Statin	8,132 (62.3%)
Current smoking, *N* (%)	1,180 (9.0%)
Estimated 10-year heart failure risk, median (IQR)	8.0% (6.2, 11.2)

### Study Protocol

**Figure 1:**
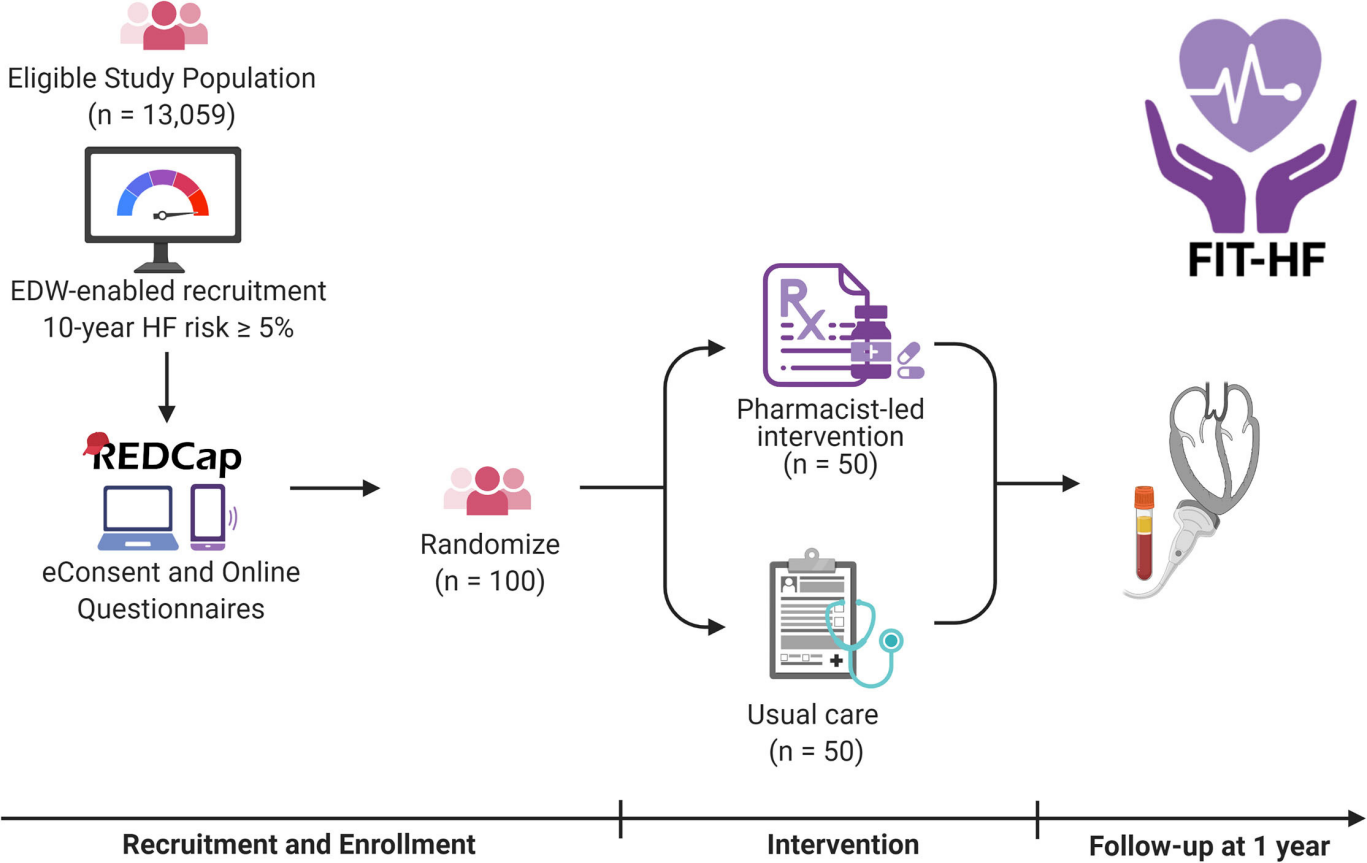
Overview of the FIT-HF study. Participants will be recruited by invitation from the eligible study population using an electronic health record-based report. They will complete eConsent and questionnaires online and have biomarkers measured and echocardiograms performed at baseline and 1 year. In the interim, they will be randomized 1:1 to a pharmacist-led intervention or to usual care. HF represents heart failure; EDW Enterprise Data Warehouse. Created with BioRender.com.

A research coordinator will call patients listed in the recruitment report to confirm eligibility, describe the study, and ask if they are interested in participating. If so, the research coordinator will review study procedures over the phone and schedule a visit in the research clinic. Participants will be emailed a link to sign electronic consent (e-Consent) and complete a baseline health and lifestyle questionnaire. The e-Consent option will offer flexibility and minimize in-person time to decrease participant burden during and after the COVID-19 pandemic. The questionnaire is composed of previously validated questions from the World Health Organization, Multi-Ethnic Study of Atherosclerosis (MESA), and EuroQol. Participants who are unwilling or unable to complete these procedures online will be instructed to arrive 30 min before their scheduled visit in order to sign written consent and complete the questionnaire in person. After consent is obtained, participants will then be randomized in 1:1 fashion to intervention vs. usual care (Figure 1). The randomization table will be generated using random permuted blocks of sizes four and six by a statistician with no other involvement in the study.

All participants will undergo phlebotomy for measurement of BNP, hs-cTn, basic metabolic panel, lipid panel, and biorepository storage, and baseline echocardiography with Doppler measurements. All participants will also undergo measurement of vital signs and receive an educational packet of information from the Centers for Disease Control and Prevention and American Heart Association on signs and symptoms of HF and heart-healthy lifestyle.

**Figure 2:**
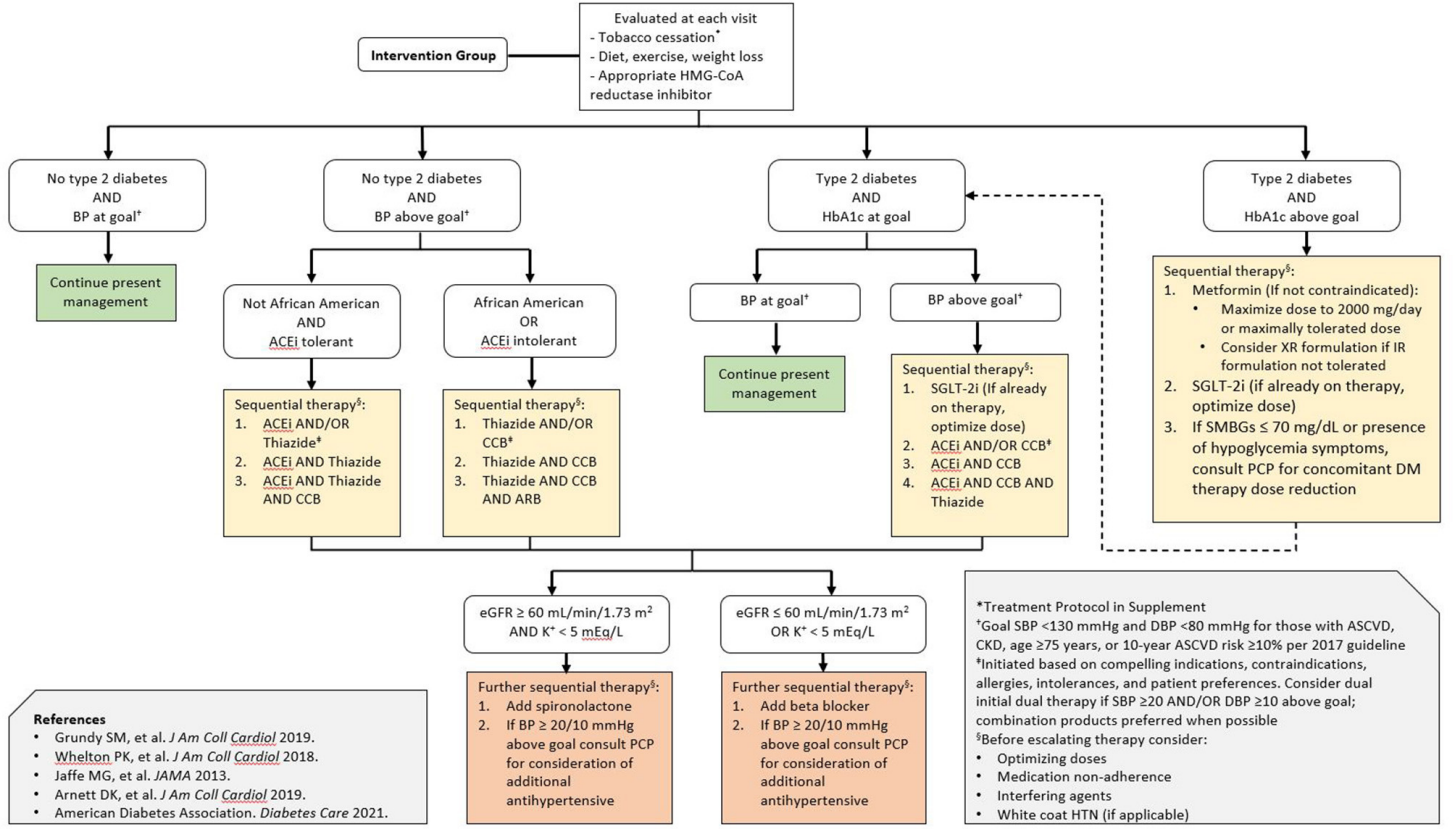
Pharmacist-directed intervention treatment algorithm. The treatment algorithm was derived from professional society guidelines for the primary prevention of cardiovascular disease as well as blood pressure, glucose, and lipid lowering. Special consideration is given to the early initiation of SGLT-2 inhibitors in patients with diabetes, given the evidence supporting their efficacy in heart failure prevention and current guideline recommendations. BP represents blood pressure; ACEi angiotensin converting enzyme inhibitor; ARB angiotensin receptor blocker; CCB calcium channel blocker; SMBG self-monitored blood glucose; eGFR estimated glomerular filtration rate; PCP primary care physician; DM diabetes mellitus; SBP systolic blood pressure; DBP diastolic blood pressure; HTN hypertension.

Participants who are randomized to the intervention arm will receive a referral to the pharmacist-led intervention (supervised by an attending cardiologist), and have their first visit on the same day as the baseline testing. Two pharmacy residents will deliver the intervention under the supervision of an attending pharmacist. Residents have received standardized training on lifestyle modification and risk factor management through the residency program. Pharmacists will review participants' medications and may make adjustments according to a medication protocol (Figure 2) for blood pressure, glucose, and lipid control according to the latest evidence-based practice guidelines. Specifically, the medication protocol specifies blood pressure control according to the protocol employed by Kaiser Permanente Health (24), consideration of SGLT-2 inhibitor initiation in patients with diabetes per ACC/AHA and American Diabetes Association (ADA) guidelines (5, 25), and statin initiation or intensification per the 2018 ACC/AHA cholesterol guideline (20). Pharmacists will additionally counsel patients regarding heart-healthy lifestyles and may order laboratory tests when medications are added to follow potassium levels, kidney function, or other relevant laboratory parameters according to the standard of care. Adherence to follow-up lab tests is monitored by the pharmacists, with reminders sent at their discretion according to their typical clinical practice. The study population comprises patients who receive their usual care on the same medical campus as the study site, mitigating access to care limitations for the purposes of this pilot study. The pharmacist visits are provided at no cost to participants; medications are e-prescribed to participants' preferred pharmacies according to the standard of care and are billed through participants' insurance. The results from participants' BNP, hs-cTn, and echocardiography will be provided to them and their primary care physicians as well as the pharmacist and cardiologist team members. Participants in the intervention arm will receive follow-up from the pharmacist team approximately every 3 months for a period of 1 year. Follow-ups will be clinical telephone visits structured similarly to the baseline visit, with interim review of laboratory values, if applicable, for medication monitoring and titration. The pharmacist team will provide a detailed note in the electronic medical record to the primary care doctor after every clinical encounter.

The participants (and their primary care physicians) who are randomized to usual care will be blinded to their 10-year heart failure risk estimates as well as biomarker and echocardiography results, unless there are critical or alert values from the hs-cTn (i.e. >500 pg/mL) or echocardiography (see Supplementary Methods), in which case their primary care physician will be informed. All patients will be requested to repeat the health and lifestyle questionnaire and schedule an in-person visit at 1-year follow-up. At this visit, all participants will undergo repeat measurement of vital signs and anthropometrics, phlebotomy, and echocardiography. The 1-year follow-up time was selected based on a limited duration by which a significant change in risk factor control would be expected based on prior studies (11, 13, 18). Participants receive free parking for all study-related visits and are paid $25 at the end of the study for their time spent participating.

Study data will be collected and managed using REDCap (Research Electronic Data Capture) electronic data capture tools hosted at Northwestern University (26, 27). REDCap is a secure, web-based software platform designed to support data capture for research studies, providing (1) an intuitive interface for validated data capture; (2) audit trails for tracking data manipulation and export procedures; (3) automated export procedures for seamless data downloads to common statistical packages; and (4) procedures for data integration and interoperability with external sources.

### Outcomes

The primary outcome is change in BNP at 1 year. Secondary outcomes include change in hs-cTn, systolic and diastolic blood pressure, weight and body mass index, serum glucose and low density lipoprotein cholesterol, estimated glomerular filtration rate and creatinine, and current smoking at 1 year. Exploratory outcomes include medication adherence and changes in echocardiographic parameters such as left ventricular mass index, diet and physical activity, and health utility at 1 year. Adherence to medications is measured using the same questionnaire as the Atherosclerosis Risk in Communities (ARIC) study: the four-item Morisky Green Levine Scale, frequency of non-adherence in the past 4 weeks, and direct measure of adherence in the past 4 weeks (28). Feasibility will also be examined by monitoring participant retention rates. Retention will be defined as completion of the 1-year study protocol and measured as the proportion of enrolled participants in each arm who successfully complete the study. All demographics (e.g., sex, race, ethnicity, education), health behaviors (e.g., smoking, diet, physical activity), and patient-centered outcomes (e.g., health-related quality of life) are self-reported.

The following serious adverse events will be reported: echocardiogram or troponin alert (at baseline or 1-year follow-up), death, hospitalization, or ER visit (all at 1-year follow-up, or sooner if the study team becomes aware of the adverse event). All adverse events will be determined by chart review and personally adjudicated by the principal investigator. As a process of care intervention using guideline-directed medical therapy (rather than a trial of a novel therapy), drug-related adverse events will not be reported for this study.

### Statistical Analysis

Baseline characteristics will be reported by group assignment in the form of means (standard deviations) and medians (interquartile ranges) for continuous variables, as appropriate, and proportions for categorical variables. The analysis will use two-sample *t*-tests to compare continuous variables and χ^2^-tests to compare categorical variables between the pharmacist-directed intervention and usual care groups. All participants will be included according to the intention-to-treat principle for all endpoints. A per protocol analysis will be reported as a secondary analysis to evaluate whether study dropout significantly influenced the outcome of the trial. Because the total study population is 100, subgroup analyses are not planned for this study due to insufficient power. Since this is a pilot and feasibility study, no formal power analysis has been performed to inform the sample size. However, given *N* = 50 in each study arm and under the assumption of similar BNP changes as in STOP-HF (7), the study would have 80% power to detect a 12 pg/mL mean difference in the change in BNP between the two study arms.

## Discussion

This report describes, to our knowledge, the first clinical study of a quantitative risk-based screening and pharmacist-led intervention for the primary prevention of heart failure. BNP-based risk assessment has shown potential to identify patients at higher risk of developing heart failure, and other biomarkers such as hs-cTn and imaging modalities such as echocardiogram have a potential role in sequential testing strategies. However, such adjunctive risk stratification methods are unlikely to be cost-effective if applied to the general population (29), and an important gap remains to understand which subpopulations of patients may be best to target for screening and intensification of preventive care. Successful completion of the FIT-HF pilot trial, leveraging the multivariable risk prediction tool, PCP-HF, is anticipated to offer insight into the feasibility, safety, and potential efficacy of a risk-based strategy to reduce the risk of incident HF events. Examination of the distributions of predicted risk, biomarkers, clinical data, and echocardiographic parameters, and how these measures respond to a pharmacist-led intervention, will support refinement of the risk-based protocol for a future well-powered, multi-center clinical outcomes trial.

Prior studies of pharmacist-led interventions for individual risk factors or chronic conditions, such as blood pressure and diabetes, have shown that pharmacist management is often superior, if not equal, to usual care (30–35). However, neither pharmacist-based intervention nor other non-physician team-based care has been tested specifically in HF prevention, in particular using the risk factor-agnostic strategy of FIT-HF. Thus, a feasibility study is warranted, and the study outcomes align with two of the eight general areas of feasibility studies as described by Bowen et al. (36): limited efficacy (evaluated using the primary outcome of change in BNP as a key intermediate variable) and practicality (evaluated with successful enrollment of 100 participants and high retention through the end of the study). As this process of care trial will test a new concept of HF prevention, usual care rather than another non-physician role has been selected as the comparator. The usual care group is selected as individuals with a primary care physician in the health system and, therefore, have access to routine care. However, an important limitation to note if there is a significant difference between the two groups is that the intervention group likely has more interactions with the healthcare system during the study period.

The ultimate goal of this research is to develop a paradigm for HF prevention that is analogous to established practice guidelines for risk-based ASCVD prevention. Even in the context of ASCVD prevention, prospective randomized trial data of quantitative risk-based interventions are limited; most prior trials have used inclusion criteria based on the presence of one or more risk factors, and quantitative risk, if used, is added as an additional inclusion criterion (37). Thus, the benefit of a pure quantitative risk-based prevention strategy is largely extrapolated from such trials and observational data. A large trial of quantitative ASCVD risk modification, the Centers for Medicare and Medicaid Services-sponsored Million Hearts Model evaluation, is underway; a pre-specified interim analysis showed improvements in the use of statins and antihypertensives (38). In this context, FIT-HF and subsequent studies may also inform the design of risk-based interventions in the primary prevention of cardiovascular diseases more generally. A key consideration to strengthen these studies to be generalizable and promote health equity is the representative recruitment of participants. Given practical constraints on the scope of a pilot study, FIT-HF will recruit from a convenience sample of patients of a large urban medical center. Although the representation of Black patients in our recruitment report is 17%, we recognize that due to socioeconomic and structural factors such as competing demands of work and residential segregation, the final recruitment of Black patients into FIT-HF may not be as high. However, we have assembled a diverse study team with expertise in recruiting populations traditionally underrepresented in clinical research. In larger, multi-centered trials that may follow, we propose partnerships with community organizations and study sites in under-resourced communities with lower access to care to ensure that the population studied represent those at greatest risk of HF, as well as use of the RE-AIM framework during a wider implementation phase to evaluate whether the intervention has the intended public health impact (39).

Of particular relevance to potential strategies for HF prevention are the SGLT-2 inhibitors. Large outcome trials have already supported the use of SGLT-2 inhibitors not only to decrease major adverse cardiovascular events in patients with HF with reduced ejection fraction (40, 41), but also to prevent HF in patients at high risk based on established risk factors of diabetes (42) and chronic kidney disease (43). In response, multiple professional societies have recommended the use of SGLT-2 inhibitors in patients with type two diabetes (5, 25), and these guideline recommendations were incorporated into the medication protocol of the FIT-HF intervention. As ongoing trials validate the efficacy of SGLT-2 inhibitors for HF prevention in other subgroups, such as those with chronic kidney disease, future guidelines may be able to broaden the patient populations in which SGLT-2 inhibitors are recommended; SGLT-2 inhibitors could potentially take a place in heart failure prevention akin to statins for atherosclerotic cardiovascular disease prevention (44). In this context, pharmacists are uniquely positioned to collaborate with physicians in identifying appropriate patients for SGLT-2 inhibitor initiation, counseling patients on their risks and benefits, and prescribing and titrating the medication. Future studies may be able to incorporate pharmacist-led intensification of preventive care in the context of expanded indications for SGLT-2 inhibitors to primary prevention patients without diabetes, given the low risk of hypoglycemia with these agents owing to their mechanisms of action (45).

## Conclusion

The FIT-HF pilot study will test a strategy of quantitative risk-based enrollment into a screening and pharmacist-led intervention program for the primary prevention of heart failure. FIT-HF is designed to be an initial step toward developing a robust, generalizable, and efficacious prevention strategy to disrupt the increasing burden of heart failure in the general population.

## Ethics Statement

The studies involving human participants were reviewed and approved by Northwestern University Institutional Review Board. The patients/participants provided their written informed consent to participate in this study.

## Author Contributions

MW, BD, LV, NM, AW, KH, AL, SS, CY, DL, and SK: conception and design. All authors: acquisition, analysis, or interpretation of data and revision of manuscript for important intellectual content. MW: drafting of manuscript. SS, CY, DL, and SK: supervision.

## Funding

Supported by grants from the American Heart Association (#19TPA34890060) and National Institutes of Health (P30DK092939 and P30AG059988) to SSK. The funding sponsor did not contribute to design and conduct of the study, collection, management, analysis, or interpretation of the data or preparation, review, or approval of the manuscript.

## Conflict of Interest

The authors declare that the research was conducted in the absence of any commercial or financial relationships that could be construed as a potential conflict of interest.

## Publisher's Note

All claims expressed in this article are solely those of the authors and do not necessarily represent those of their affiliated organizations, or those of the publisher, the editors and the reviewers. Any product that may be evaluated in this article, or claim that may be made by its manufacturer, is not guaranteed or endorsed by the publisher.
