# Corrigendum: Rationale and Design of a Pharmacist-led Intervention for the Risk-Based Prevention of Heart Failure: The FIT-HF Pilot Study

**DOI:** 10.3389/fcvm.2022.844270

**Published:** 2022-02-18

**Authors:** Michael C. Wang, Bridget Dolan, Benjamin H. Freed, Lourdes Vega, Nikola Markoski, Amy E. Wainright, Bonnie Kane, Laura E. Seegmiller, Katharine Harrington, Alana A. Lewis, Sanjiv J. Shah, Clyde W. Yancy, Ian J. Neeland, Hongyan Ning, Donald M. Lloyd-Jones, Sadiya S. Khan

**Affiliations:** ^1^Department of Preventive Medicine, Northwestern University Feinberg School of Medicine, Chicago, IL, United States; ^2^Department of Pharmacy, Northwestern Memorial Hospital, Chicago, IL, United States; ^3^Division of Cardiology, Northwestern University Feinberg School of Medicine, Chicago, IL, United States; ^4^Department of Medicine, University Hospitals Cleveland Medical Center, Cleveland, OH, United States; ^5^Case Western Reserve University School of Medicine, Cleveland, OH, United States

**Keywords:** heart failure, primary prevention, pharmacist, risk prediction, natriuretic peptides

In the original article, there was a mistake in Figure 2 **(Pharmacist-directed intervention treatment algorithm)** as published. **The decision tree boxes “African American OR ACE inh intolerant” and “Not African American AND ACE inh tolerant” were reversed, and the abbreviation “ACE inh” instead of “ACEi” was used**. The corrected Figure 2 **(Pharmacist-directed intervention treatment algorithm)** appears below.

**Figure 2:**
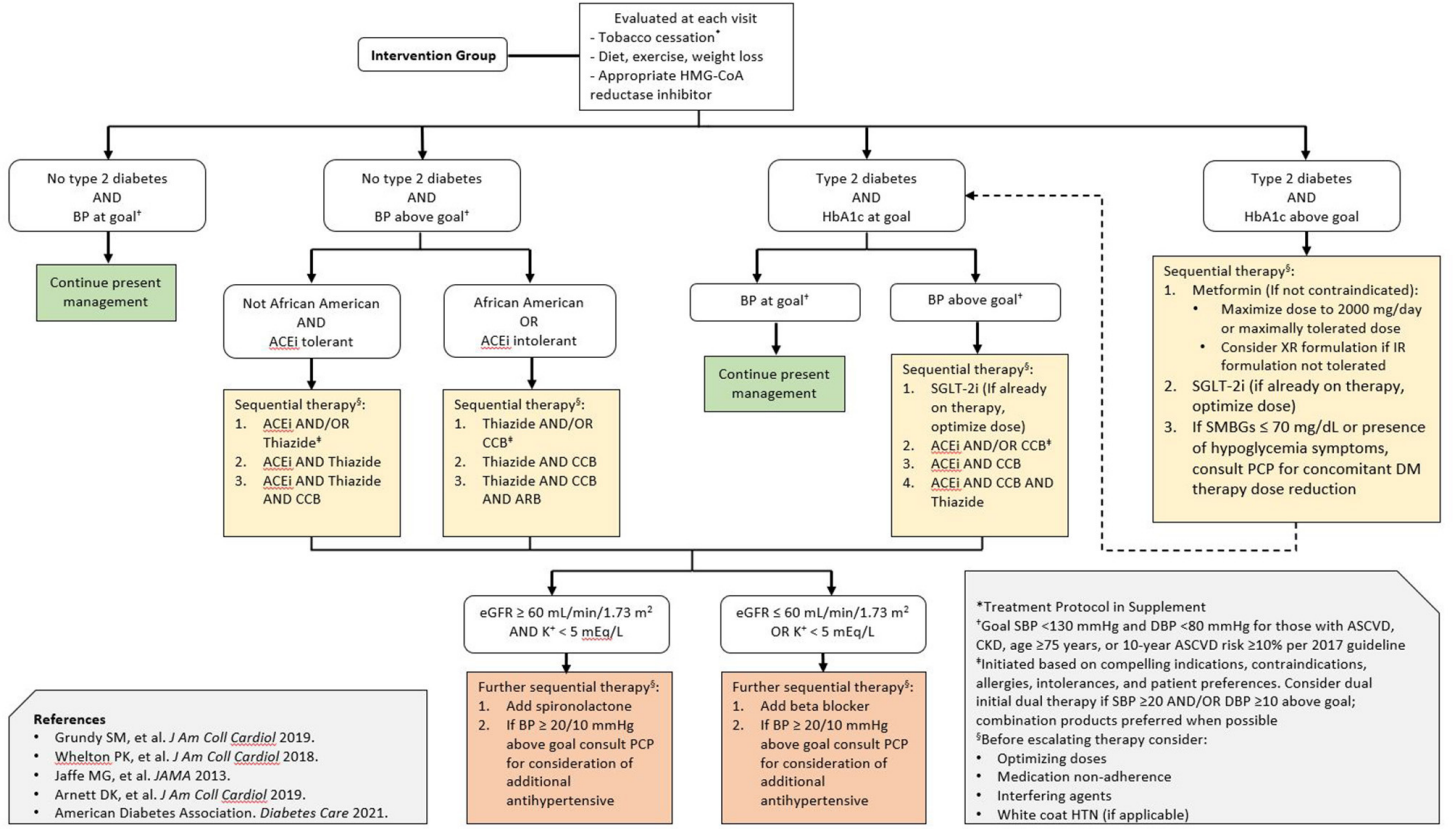
Pharmacist-directed intervention treatment algorithm. The treatment algorithm was derived from professional society guidelines for the primary prevention of cardiovascular disease as well as blood pressure, glucose, and lipid lowering. Special consideration is given to the early initiation of SGLT-2 inhibitors in patients with diabetes, given the evidence supporting their efficacy in heart failure prevention and current guideline recommendations. BP represents blood pressure; ACEi angiotensin converting enzyme inhibitor; ARB angiotensin receptor blocker; CCB calcium channel blocker; SMBG self-monitored blood glucose; eGFR estimated glomerular filtration rate; PCP primary care physician; DM diabetes mellitus; SBP systolic blood pressure; DBP diastolic blood pressure; HTN hypertension.

The authors apologize for this error and state that this does not change the scientific conclusions of the article in any way. The original article has been updated.

## Publisher's Note

All claims expressed in this article are solely those of the authors and do not necessarily represent those of their affiliated organizations, or those of the publisher, the editors and the reviewers. Any product that may be evaluated in this article, or claim that may be made by its manufacturer, is not guaranteed or endorsed by the publisher.

