# Supplementary material for: Rationale and Design of a Pharmacist-led Intervention for the Risk-Based Prevention of Heart Failure: The FIT-HF Pilot Study
Source: Front Cardiovasc Med. 2021 Nov 29;8:785109. doi: 10.3389/fcvm.2021.785109 (PMC8667267; doi:10.3389/fcvm.2021.785109)
Supplement: Supplementary file 1 [file Data_Sheet_1.DOCX]

**Rationale and design of a pharmacist-led intervention for the risk-based prevention of heart failure: the FIT-HF pilot study**

**SUPPLEMENTAL MATERIAL**

**SUPPLEMENTAL METHODS**

**Inclusion and Exclusion Criteria**

Inclusion criteria include:

1. Age 30-79 years
2. At least one internal medicine office visit to a practice on the Northwestern Medicine central campus within the past year
3. At least two such office visits within the past 5 years
4. Total cholesterol, HDL cholesterol, and glucose values available within the past 5 years
5. Predicted 10-year risk of heart failure ≥5% based on the Pooled Cohort Equations to Prevent Heart Failure (PCP-HF) and most recently available data

Exclusion criteria include:

1. History of cardiovascular disease, excluded based on International Classification of Diseases, 9^th^ and 10^th^ Revision (ICD 9 and ICD 10) codes and verbally confirmed with participants
   1. Stroke/Cerebrovascular disease
      1. ICD 9: 430-432.9; 433.x; 434.x
      2. ICD 10: I60-62; I63.x; I65.x; I66.x
   2. Coronary artery disease (including myocardial infarction and acute coronary syndrome)
      1. ICD 9: 410.x; 411.x; 412; 413.x; 414.x
      2. ICD 10: I20.xx; I21.x; I22.x; I23.x; I24.8; I24.9; I25.x
   3. Peripheral artery disease
      1. ICD 9: 440.2x; 440.3x; 443.9x
      2. ICD 10: I70.2x; 170.3x; 170.4x; 170.5x; 170.6x; 170.7x; I73.9x
   4. Heart failure
      1. ICD 9: 402.x1; 404.x1; 404.x3; 428.x
      2. ICD 10: I09.81; I11.0; I13.0; I50.x
   5. Atrial fibrillation
      1. ICD 9: 427.31; 427.32
      2. ICD 10: I48.x
   6. Pacemaker
      1. ICD 9: V45.x
      2. ICD 10: Z95.x
2. Clinical signs or symptoms of heart failure
3. Current pregnancy
4. Estimated glomerular filtration rate less than 45 mL/min/1.73m^2^ using the CKD-EPI (2009) formula
5. Stage 3 or 4 cancer
6. Unreachable by telephone (no telephone number on file)
7. No identifiable electronic health record (missing medical record number)

**Echocardiogram Alert Criteria (adapted from the echocardiogram protocol of the Multi-Ethnic Study of Atherosclerosis)**

If any of the following alerts are found during a study echocardiogram, the site sonographer will contact the echocardiogram co-investigator and principal investigator, and the relevant protocol will be followed, including unblinding of results if the participant is in the control group.

**Immediate referrals** are medical emergencies in which the principal investigator reaches out to the participant’s primary care physician before the end of the participant’s study appointment, and the participant is advised to seek medical care immediately at a physician’s office or hospital. Abnormalities classified as immediate referrals include:

1. Suspected tamponade
2. Aortic aneurysm (measuring >5.0 cm) or dissection
3. Intracardiac abscess or obvious vegetation
4. Intracardiac thrombus or mass
5. Pseudoaneurysm
6. Significant arrhythmia (eg atrial fibrillation with heart rate > 110 bpm, sustained ventricular arrhythmias, or nonsustained ventricular tachycardia > 10 beats)

**Urgent referrals** are abnormalities which may require medical attention but not on an emergency basis. The principal investigator will communicate the findings to the participant and their primary care physician within one week. Abnormalities classified as urgent referrals include:

1. Severe left ventricular or right ventricular enlargement
2. Severe stenosis of any valve
3. Severe regurgitation of any valve
4. Moderate or greater pericardial effusion without evidence of tamponade
5. Atrial fibrillation with heart rate < 110 bpm

**Alerts** are medical findings that may have adverse health consequences to the participant if left untreated. The principal investigator has discretion as to when to disclose alerts to the participant and their primary care physician based on the findings, patient history, and clinical context. Abnormalities classified as alerts include:

1. Mild or moderate stenosis of any valve
2. Moderate mitral or aortic regurgitation
3. Moderate or greater dynamic left ventricular outflow tract obstruction (gradient at rest or with Valsalva)
4. Intra-cardiac shunt
5. Moderate to severe pulmonary hypertension (right ventricular systolic pressure > 45 mmHg)
6. Evidence of right ventricular pressure or volume overload
7. Low ejection fraction (<40%) or wall motion abnormality

**SMOKING CESSATION PROTOCOL**

**Supplemental Figure 1: Smoking Cessation Treatment and Monitoring Algorithm**

**Supplemental Figure 2. Pharmacotherapy Initiation and Titration Protocol**

**References:**

1. 2018 American College of Cardiology Expert Consensus Decision Pathway on Tobacco Cessation Treatment (Barua R, Rigotti N, et al. *Journal of the American College of Cardiology*. 2018; 72(25):3332-65).
